# Off‑label and unapproved pediatric drug utilization: A meta‑analysis

**DOI:** 10.3892/etm.2024.12701

**Published:** 2024-08-30

**Authors:** Xingxing Yuan, Jiawei Gao, Liuxin Yang, Yurong Tan, Ousman Bajinka

**Affiliations:** 1 Department of Gastroenterology, Heilongjiang Academy of Traditional Chinese Medicine, Harbin, Heilongjiang 150006, P.R. China; 2 First Clinical Medical College, Heilongjiang University of Chinese Medicine, Harbin, Heilongjiang 150040, P.R. China; 3 Department of Medical Microbiology, Central South University, Changsha, Hunan 410078, P.R. China

**Keywords:** off-label prescriptions, licensed drug, unlicensed prescribing, pediatrics

## Abstract

Despite legislative enforcement on authorized drugs, off-label and unapproved pediatric drug use is prevalent. The present study aimed to assess the global prevalence of off-label and unlicensed prescriptions among hospitalized children via meta-analysis. A comprehensive examination of articles published between 1990 and 2023 from the PubMed, Scopus, Excerpta Medica Database, Web of Science and Google Scholar databases was conducted. Key word-based advanced searches were executed using the aforementioned databases. A total of 45 studies that reported the prescriptions of off-label and unlicensed drugs to pediatric patients were included. The global prevalence of off-label and unlicensed drug prescriptions to children in pediatrics or neonatal departments was 56%. Patient sample sizes varied from 40-13,426, with a range of 240-8,891 total prescriptions issued. Of the 45 studies examined, 22 studies originated from Europe, 13 from Asia, 3 from South America, 3 from Africa, and 2 each from North America and Australia. Africa had the highest prevalence rate at 66%, followed by Asia, South America, North America, Australia and Europe. The present meta-analysis demonstrated that the prevalence of off-label and unlicensed drug prescriptions given to pediatric patients was notably high and geographically diverse. Therefore, drug authorities should standardize pediatric prescription practices in future.

## Introduction

An unapproved prescription describes the provision of a medicine without marketing authorization for human use, whereas an off-label prescription describes the use of an authorized drug outside of the recommended age group, indication, dosage, route of administration or frequency of use (1). Pharmaceutical trials of medicines that are not tested in children may result in off-label or unapproved drug prescriptions provided to children (2). Despite legislative enforcement on authorized drugs, approved pediatric formulations are ineffective for the pharmaceutical industry (3). A function of the Mutual Recognition Agreements signed by The European Union (EU) and third-country authorities is to assess drug safety and efficacy of medicines prior to public release (4). However, testing of drug safety and efficacy in children raises ethical concerns, and therefore, the number of pediatric clinical trials is limited (5,6). This may lead to clinicians prescribing drugs to children using data from adult clinical trials, which could lead to increased numbers of unapproved and off-label prescriptions provided to children (7). Off-label and unauthorized prescriptions present significant risks to children (8). However, a previous study reported that, within a 2-month period of observation, the prevalence of off-label and unlicensed drug use was increased in both pediatric intensive care units (PICUs) and neonatal intensive care units (NICUs), compared with a study at the same location 16 years prior, despite the anticipated impact of current regulatory efforts (9).

As of early 1937, 353 patients were exposed to Elixir sulfanilamide. Nearly one-third of them, 34 children and 71 adults, soon died of acute kidney failure (10). A total of 25 years later, use of the over-the-counter sleeping pill thalidomide was reported to induce teratogenesis in consumers in America, Australia and Europe (11). Furthermore, in 1971, diethylstilbestrol (a non-steroidal estrogen medication), 30 years after its approval by the Food and Drug Administration, was discontinued due to its association with breast cancer (12). These occurrences necessitated stringent drug regulations to ensure efficacy of approved drugs and safeguard the health of their users. The prescription of off-label and unlicensed drugs to children may potentially be associated with toxicity and adverse drug effects (13). A specific condition that may be associated with the use of off-label and unlicensed drugs in children is bipolar depression in depressive episodes. Currently available antidepressants in children are unsatisfactory; for example, a number of systematic reviews have indicated that antidepressant methylphenidate and memantine have high non-response rates and non-tolerance (14). High non-response rates and non-tolerance in children are also associated with second-generation antipsychotic drugs such as amisulpride, clozapine, olanzapine and risperidone (15).

Differences in use of off-label and unauthorized prescriptions between countries may be attributed to varying stringency of drug regulatory authorities and pharmaceutical spending budgets, which are associated with gross domestic product (GDP) (16). The lack of pediatric-specific medications may contribute to off-label and unlicensed prescriptions. Multi-collaborative efforts involving researchers, medical staff, industry regulators and policy makers may be required to establish pediatric formulations in the future. To the best of our knowledge, this is the first study to estimate the global pooled prevalence of off-label and unlicensed prescriptions given to hospitalized children in NICUs, PICUs or standard pediatric wards, using meta-analysis.

## Materials and methods

### Literature search strategy

The present systematic review adhered to the preferred reporting items for systematic reviews and meta-analyses (PRISMA) guidelines (17). A comprehensive literature search was conducted on January 12, 2023 using the PubMed (https://pubmed.ncbi.nlm.nih.gov/), Scopus (https://www.elsevier.com/zh-cn/products/scopus/search), Web of Science (https://www.webofscience.com/wos) and Google Scholar databases (https://scholar.google.com/) for articles published between 1990 and 2023. Medical subject headings and key word queries were used across all databases. The PubMed database was searched using the key words, ‘off label use’ and ‘prevalence’, in combination with ‘children’. The other databases used queries such as ‘prevalence’, ‘off label drug use’ or ‘unlicensed drug use’, alongside ‘children’, with the language preference set to English and document type set to articles. Only observational studies that reported prevalence of off-label or unlicensed prescribing in hospitalized pediatric patients and published in English were included. Reviews, duplicates and non-full text articles were excluded.

### Data extraction

Data search and extraction was conducted by two independent researchers using EndNote (version X9; Clarivate Plc.). The researchers independently selected the most relevant articles using the titles and abstracts of articles. Full-text articles were selected and assessed for eligibility. An independent third party arbitrated discrepancies. Data were extracted from each article using a standardized form with the following fields: Author, year, country, mean age, prevalence of off-label and unlicensed drug use, study design, sample size, study setting, total prescriptions, prescription drugs, and off-label and unlicensed prescriptions.

### Risk of bias and quality assessment

The Joanna Briggs Institute critical assessment checklist (18) was used to assess the quality of included studies, which included the likelihood of bias in design, implementation and analysis (Table SI). The checklist has nine questions, answers to which are either yes, no, unclear or not applicable. High quality studies achieve a minimum of 5 affirmative responses.

### Statistical analysis

The primary outcome of the present study was prevalence of off-label and unlicensed prescriptions to children. The following analyses were conducted: Pooled prevalence of off-label prescriptions among i) ICU and ii) general pediatric ward patients, and pooled prevalence of unlicensed prescriptions among iii) ICU and iv) general pediatric ward patients. The prevalence of off-label or unlicensed prescriptions was calculated by dividing the number of off-label or unlicensed prescriptions by total number of prescriptions. STATA (version 16; StataCorp LP) software was used to calculate SE and CI values, and for forest map generation. The random-effects model was used regardless of the outcome of the heterogeneity analysis. I^2^>50% was considered to indicate high heterogeneity. Subgroup analysis was conducted using data from the International Monetary Fund on global GDP distribution (16). Sensitivity analysis was performed by the exclusion of individual studies one at a time and those of insufficient quality were excluded. Egger's test and funnel plots were used to evaluate publication bias. The χ^2^ test was used for categorical data. P<0.05 was considered to indicate a statistically significant difference.

## Results

### Literature search and screen

**Figure 1:**
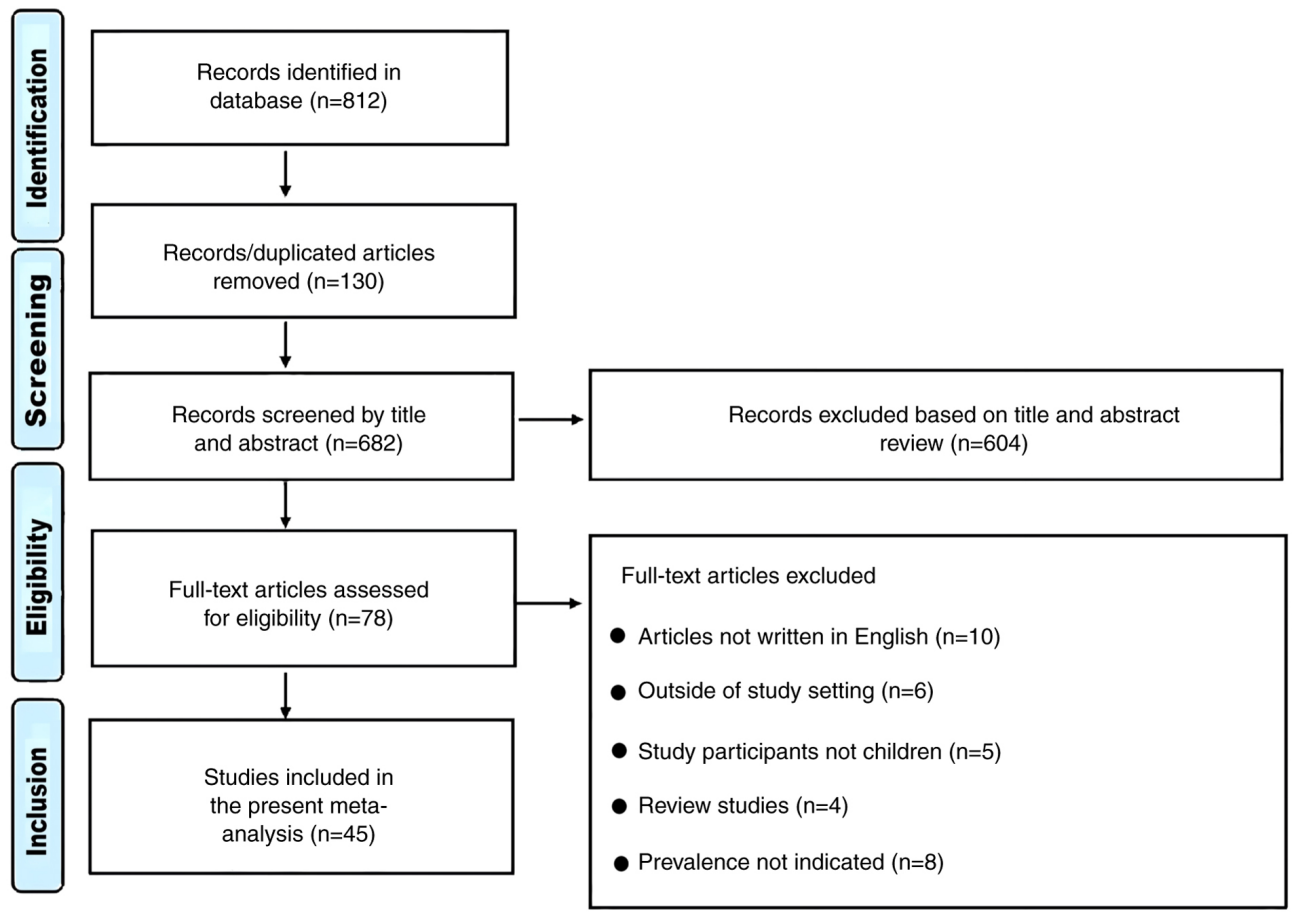
Preferred reporting items for systematic reviews and meta-analyses flowchart of the inclusion process.

A total of 812 articles were examined, of which 130 duplicates were excluded (Fig. 1). Subsequently, 604 articles were excluded following examination of the title, full text and abstract, which left 78 studies. Further exclusions included 10 studies that were not written in English, 8 that did not report on prevalence, 6 studies that were conducted outside the NICU, PICU or general pediatric ward, 4 reviews and 5 studies that involved mixed participants (adults and children). A total of 45 studies were included in the present study (9,10,19-61).

### Study characteristics

The 45 articles included in the meta-analysis included 26 prospective, 7 retrospective and 12 cross-sectional studies (Table I). Patient sample size varied from 40 to 13,426, with a range of 240-8,891 prescriptions issued. Of the 45 studies, 22 originated from Europe, 13 from Asia, three each from South America and Africa and 2 each from North America and Australia. Off-label prevalence was reported in all articles (10.0-87.0%), while 34 studies reported the prevalence of unlicensed prescriptions (1.3-79.0%). In total, 34 (75.6%) studies reported prevalence of both off-label and unlicensed medication prescriptions among participants. All studies recruited participants from either the NICU, PICU or general ward, with 23 studies conducted in general pediatric wards. The highest prevalence of off-label drug use was reported in Estonia (87%), followed by Ethiopia (75%). The lowest off-label prevalence was in Italy (9%), followed by Zimbabwe (10%). The highest unlicensed drug prevalence was reported in Indonesia (79.0%).

### Overall pooled prevalence of off-label and unlicensed drug use

**Figure 2:**
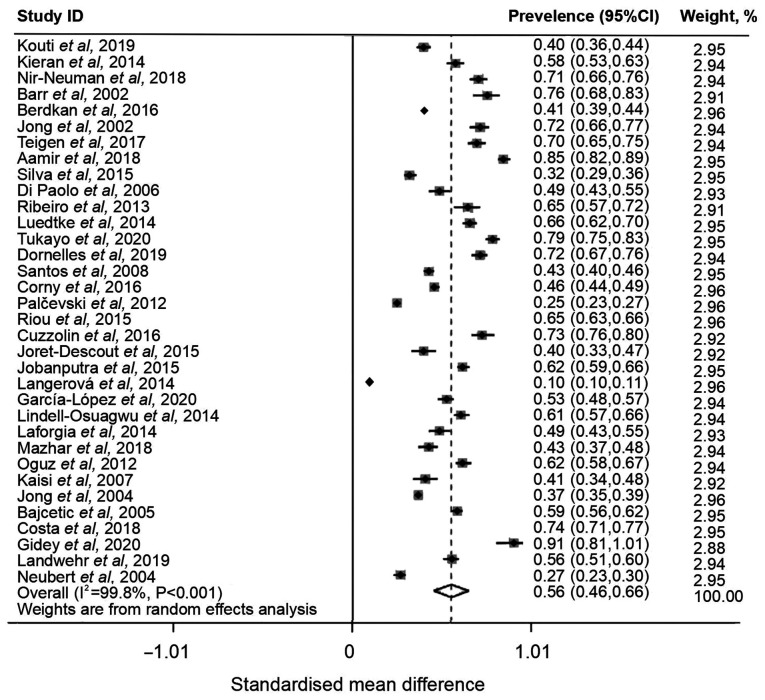
Forest plot of prevalence of off-label and unlicensed prescriptions in a pediatric population.

A total of 45 articles were included in the meta-analysis, which involved 60,997 off-label and unlicensed drugs prescribed to patients in the NICU, PICU or general wards. Due to the number of study designs used, a random-effect meta-analysis model was used to assess total number of prescriptions, and off-label and unlicensed prescriptions issue. The global prevalence of off-label and unlicensed prescriptions among pediatric or neonatal patients was 56% (95% CI, 0.46-0.66), with high heterogeneity (I^2^=99.8%; Fig. 2). As significant heterogeneity was observed, sensitivity analysis was performed. Sensitivity analysis indicated that the pooled prevalence remained notably stable upon exclusion of each study (Fig. S1). Sensitivity analysis was performed to assess the impact of excluding studies that had a small sample size of prescriptions. Following the exclusion of 12 studies that had <1,000 prescriptions, the overall combined prevalence decreased to 55% and demonstrated high heterogeneity (I^2^=99.4%; Figs. S2 and S3). The exclusion of articles with a small sample size did not significantly alter the global prevalence of off-label and unlicensed drug prescriptions to children. The high heterogeneity of included studies may be due to methodological differences of study designs. The exclusion of studies with a high risk of bias did not significantly alter the prevalence rates of off-label and unlicensed drug prescriptions (Fig. S1, Fig. S2 and Fig. S3) to children, as calculated from the sensitivity analysis .

### Overall pooled prevalence of off-label or unlicensed drug use among pediatric patients

**Figure 3:**
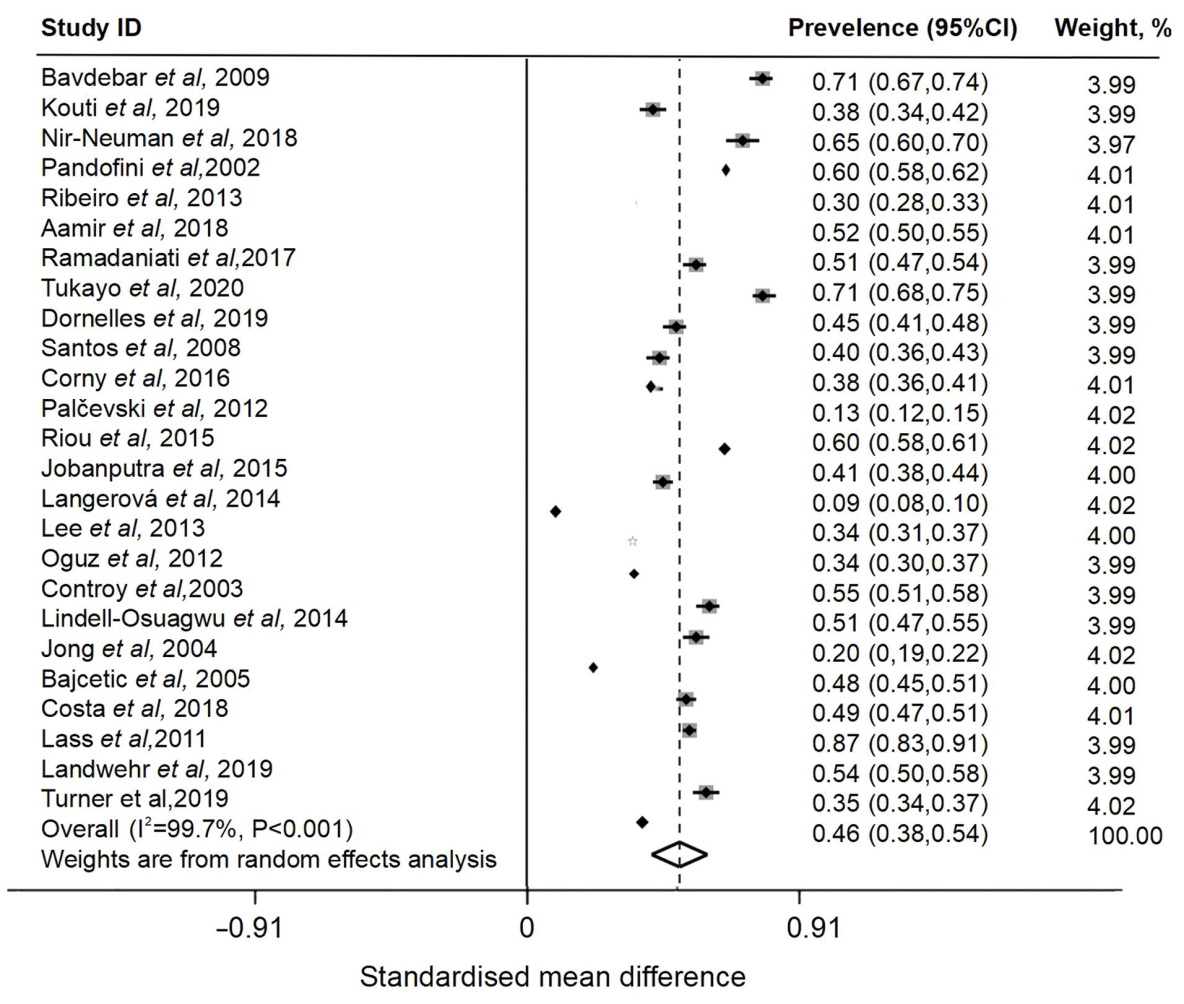
Forest plot of off-label prescriptions in pediatric patients.

**Figure 4:**
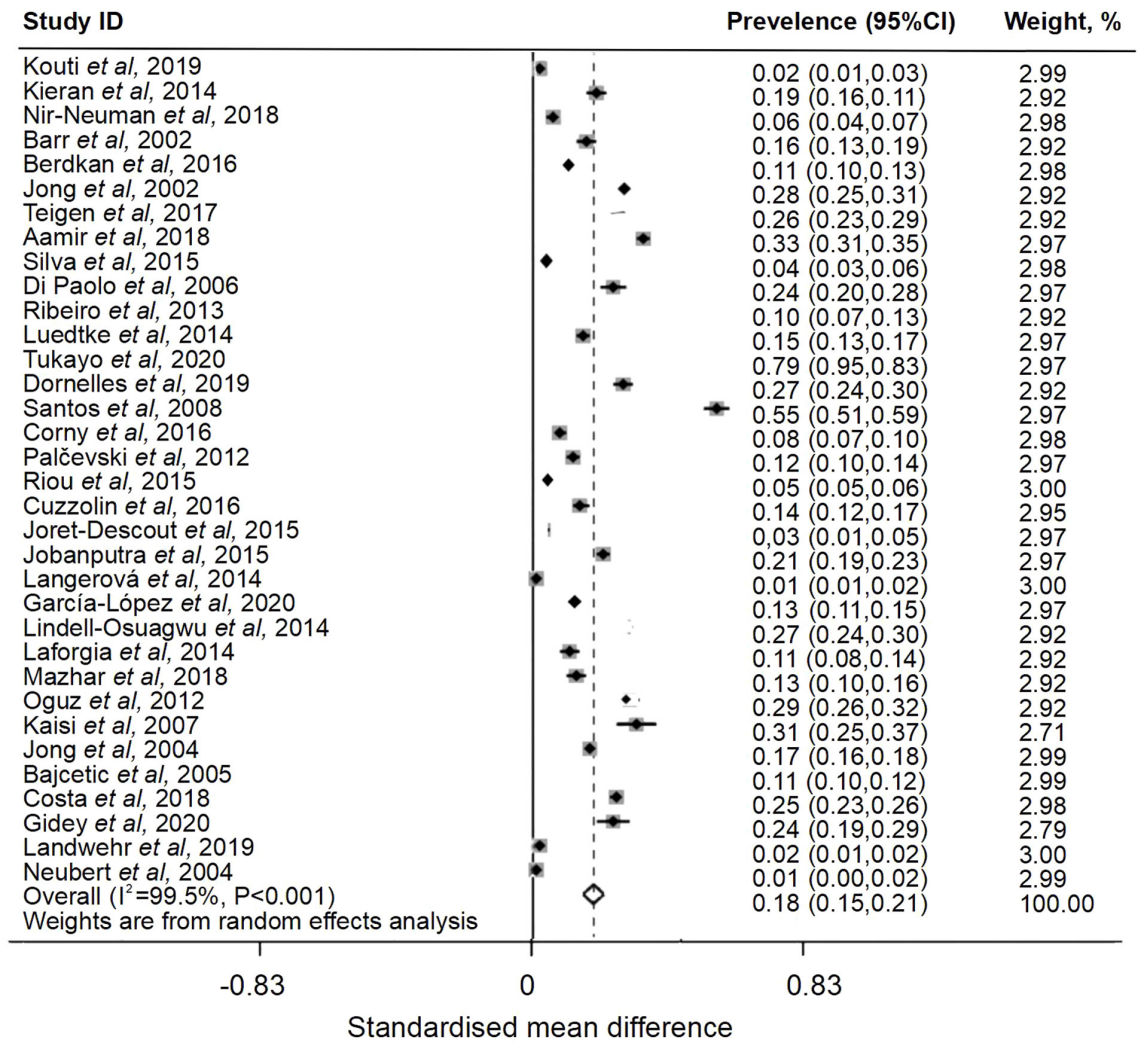
Forest plot of prevalence of unlicensed medication use among pediatric patients.

The prevalence of off-label prescriptions given to admitted pediatric patients was 46% (95% CI, 0.38-0.54), which was decreased compared with overall prevalence and had high heterogeneity (I^2^=99.7%) (Fig. 3). Of 45 included articles, 34 reported unlicensed drug usage in hospitalized children. A total of 10,126 prescriptions were unlicensed in the included articles. The global pooled prevalence rate was 18% (I^2^=99.5%; 95% CI, 0.15-0.21; Fig. 4).

### Subgroup analysis of prevalence of off-label and unlicensed drug use according to the continent of study

The subgroup analysis determined the combined prevalence of off-label and unlicensed drugs used on each continent. Africa had the highest prevalence rate at 66% (95% CI, 0.17-1.15), followed by Asia at 65% (95% CI, 0.52-0.78), South America at 63% (95% CI, 0.42-0.84), North America at 56% (95% CI, 0.36-0.76), Australia at 56% (95% CI, 0.51-0.60) and Europe at 49% (95% CI, 0.36-0.62) (Table SII).

Subgroup analysis showed that Australia had the highest prevalence of off-label drug use among pediatric patients at 54% (95% CI, 0.50-0.58), followed by Asia at 52% (95% CI, 0.42-0.63), South America at 45% (95% CI, 0.39-0.50), Europe at 42% (95% CI, 0.29-0.55) and North America at 38% (95% CI, 0.36-0.41) (Table SII). The prevalence of unlicensed medication prescriptions among pediatric patients in South America was the highest at 36% (95% CI, 0.20-0.51), followed by Africa at 27% (95% CI, 0.20-0.34), Asia at 25% (95% CI, 0.16-0.34), Europe at 12% (95% CI, 0.09-0.15), North America at 8% (95% CI, 0.06-0.09) and Australia at 2% (95% CI, 0.02-0.03) (Table SII).

### Publication bias

**Figure 5:**
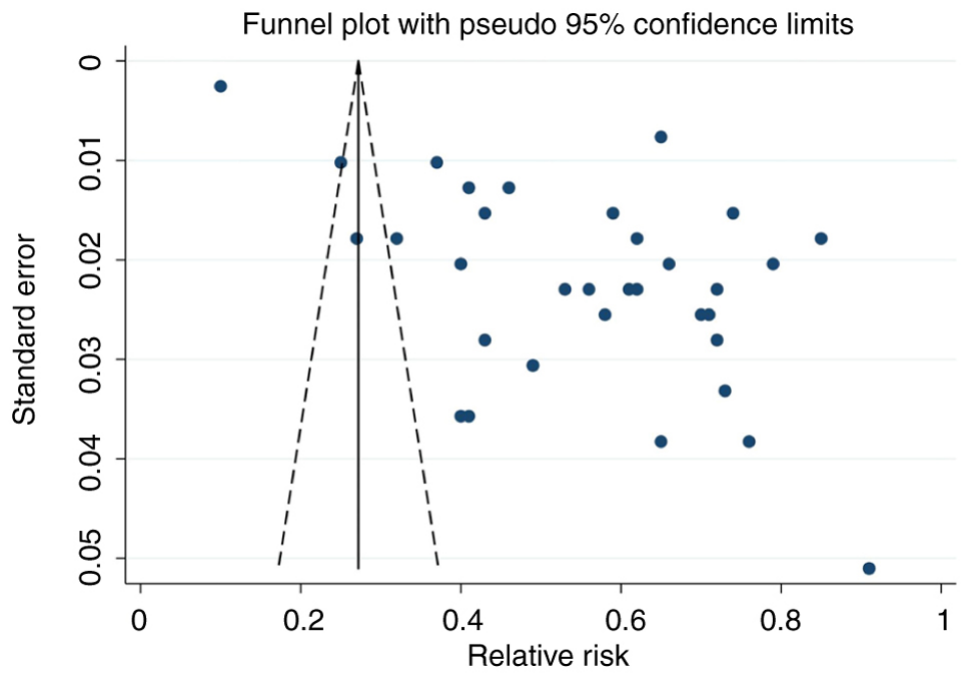
Funnel plot of studies examining off-label and unlicensed prescriptions.

Funnel plot analysis indicated notable publication bias based on the asymmetric shape of the funnel (Fig. 5). The range of effect sizes for most studies was between 0.40 and 0.80, with a low standard error. The regression-based Egger's test was significant (P=0.017), which indicated heterogeneity and publication bias (Fig. S4). Non-parametric trim-and-fill analysis identified and corrected funnel plot asymmetries in studies reporting off-label and unlicensed prescribing. Despite a degree of publication bias, the results of the present study could still be considered robust.

## Discussion

As available standardized pediatric prescription data are limited, off-label and unlicensed medications are frequently prescribed to pediatric patients. The prescription of off-label and unlicensed drugs has been escalating among children recently, and current regulations for the use of prescription medications are considered ineffective in the pharmaceutical industry (62). The present study aimed to assess the global prevalence of off-label and unlicensed drug prescriptions given to hospitalized children through meta-analysis. All prescriptions in the included studies originated from data obtained from patients that were admitted to the NICU, PICU or general ward. Of 45 studies, 34 reported the prevalence of prescriptions of both off-label and unlicensed medication given to children. The overall global prevalence of off-label and unlicensed medications among patients in the PICU or NICU was 56%. The highest prevalence was in Asia (62%), followed by South America (53%) and Europe (29.9%).

Following the exclusion of studies following sensitivity analysis, the combined prevalence of off-label and unlicensed medication use was 55% compared with an overall global prevalence of 56% in all included studies. A previous meta-analysis of 6 studies reported that off-label prescriptions constituted 93.5% of all medications prescribed (63); however, the incidence of unlicensed medications was only 3.9%. A large proportion of medications were prescribed to children <2 years of age in the aforementioned meta-analysis (63). The present study demonstrated that the prevalence of off-label prescriptions given to admitted pediatric patients was 46% and that of unlicensed prescriptions was 18%. A previous study demonstrated that the frequency of off-label medication use to children was 14.5-35%, although no meta-analysis or statistical analysis was performed (64). Differences between the aforementioned study and the present study may be attributed to different sample sizes and examination of antipsychotic drugs specifically in the previous study, whereas the present study examined all types of prescribed drug. Differences could also be attributed to differences in drug regulatory standards and pharmaceutical budgets between different countries (13,14). Subgroup analysis according to continent of study indicated the overall pooled prevalence of off-label and unlicensed drugs used Africa had the highest prevalence rate at 66%, followed by Asia at 65%, South America at 63%, North America at 56%, Australia at 56% and Europe at 49%. As high heterogeneity was demonstrated across the studies in the present meta-analysis, further studies on the prevalence of off-label and unlicensed prescriptions to hospitalized children are necessary.

A standardized definition of off-label and unlicensed medications may be required to accurately assess their frequency of use, risk factors and impact. Further prospective clinical studies should evaluate the efficacy and safety of these medications in the pediatric population. The prescription of off-label and unlicensed drugs in pediatric primary healthcare necessitates further investigation of their use by regulatory agencies and the pharmaceutical industry. The elimination of the prescription of off-label and unlicensed drugs may potentially increase drug safety in children. In the future, clinicians could report suspected adverse drug reactions, such as unipolar depression, to relevant authorities to increase drug safety in children.

The prescription of off-label and unlicensed drugs may expose children to drug toxicity and adverse drug effects, due to the pressure on clinicians to treat critically ill neonatal patients. In addition to empirical treatment methods (the doctor's treatment experience in the course of use), implementation of enhanced safe drug usage is imperative. Collaborative engagement between the pharmaceutical industry, clinical academics, healthcare professionals, parents and national regulatory bodies is key to prevent children from becoming 'therapeutic orphans'. To decrease the risk of drug misuse in the pediatric population, pharmacists should review medication charts. This, as well as drug labeling (including patient and physician instructions), is an effective method to avoid drug misuse (65). Strict supervision of drug administration can also avoid adverse drug reactions. To develop pediatric formulations, multi-collaborative efforts of researchers, medical staff, industry regulators and policymakers are required. Further evidence to support the safety and efficacy of off-label drug prescriptions to children and communication with legal guardians about proposed treatment is essential in the future.

The present meta-analysis analyzed studies that reported on the prevalence of off-label and unlicensed prescriptions among pediatric patients, and had varying study sample sizes, durations and designs. Different countries may require distinct licensing information and drug package-leaflet data. Additionally, only 2 studies each originated from North America and Australia, which limited the scope of the present study; therefore further research on this topic in North America and Australia is warranted. These limitations potentially contributed to the heterogeneity observed in the present analysis. To address this high heterogeneity, the present study investigated the source of heterogeneity, conducted sensitivity analysis and used the random-effects model. In addition, PRISMA was used to minimize reporting bias. To the best of our knowledge, the present study is the first to report the pooled prevalence of off-label and unlicensed prescriptions in hospitalized pediatric patients.

In conclusion, the present meta-analysis demonstrated that the prevalence of off-label and unlicensed prescriptions among pediatric patients was substantial and varied across geographical regions. The differences between studies may be attributed to methodological discrepancies and differences in licensing and drug leaflet information across countries; therefore, it could be recommended that drug authorities standardize pediatric prescription practices in future.

## Supplementary Material

Sensitivity analysis indicating that the pooled prevalence remains notably stable upon exclusion of each study.

Sensitivity analysis to assess the impact of excluding studies that had a small sample size of prescriptions.

Forest plot of prevalence of off-label and unlicensed prescriptions in a pediatric population after excluding studies that had a small sample size of prescriptions.

Regression-based Egger's test examining off-label and unlicensed prescriptions.

Quality assessment of included articles using the Joanna Briggs Institute critical assessment checklist.

Subgroup analysis by study design and continent.

## Figures and Tables

**Table I:** Characteristics of included studies.

			Prevalence, %					Number		
First author, year	Country of residence	Patient age	Off-label	Unlicensed	Study design	No. of patients	Study setting	Total prescriptions	Off-label and unlicensed	(Refs.)
Turner *et al*, 1999	UK	1.0 y^a^	35.3	-	Prospective	936	PICU	4,455	1,574	(19)
Conroy *et al*, 1999	UK	26.0-36.0 w	54.7	9.9	Prospective	70	NICU	455	294	(20)
Pandofini *et al*, 2002	Italy	3.7 y^b^	60.0	-	Prospective	1,461	General ward	4,255	2,547	(21)
Barr *et al*, 2002	Israel	<28.0 d	59.0	16.0	Prospective	105	NICU	525	397	(22)
Jong *et al*, 2002	Netherlands	16.7 y^a^	43.0	28.0	Prospective	293	General ward	1,017	728	(23)
Conroy *et al*, 2003	UK	-	54.6	-	Prospective	51	PICU	1,574	859	(24)
Jong *et al*, 2004	UK	8.7 y^b^	20.3	16.8	Retrospective	13,426	General ward	5,253	1,947	(25)
Neubert *et al*, 2004	Germany	<18.0 y	26.4	1.3	Prospective	178	General ward	740	198	(26)
Bajcetic *et al*, 2005	Belgrade	4.0 h -18.0 y	48.0	11.0	Prospective	544	General ward	2,037	1,202	(27)
Di Paolo *et al*, 2006	Sweden	3.0-14.0 y	25.0	24.0	Prospective	60	General ward	483	236	(28)
Kaisi *et al*, 2007	Zimbabwe	1.0-5.0 y	10.0	31.0	Prospective	300	General ward	300	123	(29)
Santos *et al*, 2008	Brazil	2.0 y^b^	39.6	55.0	Prospective	272	General ward	1,450	623	(30)
Bavdekar *et al*, 2009	India	3.6±3.7 y^b^	70.6	-	Prospective	300	PICU	2,237	1,579	(31)
Lass *et al*, 2011	Estonia	<28.0 d	87.0	-	Prospective	490	NICU	1,981	1,723	(32)
Palčevski *et al*, 2012	Croatia	6.8 m^b^	13.3	11.9	Cross-sectional	691	General ward	1,643	412	(33)
Oguz *et al*, 2012	Turkey	32.5±4.7 w^b^	33.5	28.8	Prospective	464	NICU	1,315	819	(34)
Ballard *et al*, 2013	Australia	2.6 y^b^	36.0	-	Retrospective	300	General ward	887	283	(35)
Kieran *et al*, 2014	Ireland	35.0 w^b^	39.0	19.0	Prospective	110	NICU	900	522	(36)
Silva *et al*, 2015	Portugal	36.1±4.0 w^b^	27.9	4.4	Cross-sectional	218	NICU	1,011	326	(37)
Lee *et al*, 2013	Malaysia	2.0 y^b^	34.1	27.3	Prospective	168	PICU	1,295	795	(38)
Ribeiro *et al*, 2013	Portugal	6.2±4.9 y^b^	32.2	-	Retrospective	700	General ward	724	233	(39)
Lindell-Osuagwu *et al*, 2014	Finland	1.0-12.0 y	51.0	-	Prospective	123	NICU	1,054	538	(40)
Laforgia *et al*, 2014	Italy	37.0 w^b^	37.4	11.4	Cross-sectional	126	NICU	483	236	(41)
Langerová *et al*, 2014	Italy	1.0-15.0 y^b^	9.0	1.3	Retrospective	4,282	General ward	8,559	879	(42)
Luedtke *et al*, 2014	USA	3.0 w-15.0 y	57	-	Prospective	40	General ward	240	136	(43)
Riou *et al*, 2015	France	34.0 w^b^	59.5	5.2	Prospective	910	NICU	8,891	5,752	(44)
Joret-Descout *et al*, 2015	France	5.1 y^b^	36.5	3.2	Cross-sectional	120	General ward	315	125	(45)
Jobanputra *et al*, 2015	India	6.3±1.7 w^b^	41.3	21.0	Prospective	482	PICU	1,789	1,114	(46)
Berdkan *et al*, 2016	Lebanon	3.5 y^b^	30.2	11.1	Retrospective	500	PICU	2,054	848	(47)
Cuzzolin *et al*, 2016	Italy	3.3 w^b^	59.0	14.5	Cross-sectional	220	NICU	720	529	(48)
Corny *et al*, 2016	Canada	10.9 y^b^	38.2	8.3	Cross-sectional	308	General ward	2,145	997	(49)
Ramadaniati *et al*, 2017	Indonesia	2.0 y^b^	50.8	15.1	Retrospective	67	General ward	1,553	1,023	(50)
Tefera *et al*, 2017	Ethiopia	4.5±4.3 y^b^	75.8	-	Prospective	243	General ward	800	607	(51)
Teigen *et al*, 2017	Norway	0.0-17.0 y	44.0	26.0	Cross-sectional	179	General ward	930	650	(52)
Nir-Neuman *et al*, 2018	Israel	33.0-38.0 w	64.8	5.9	Prospective	134	NICU	1,069	756	(9)
Costa *et al*, 2018	Brazil	2.4±4.4 w^b^	49.3	24.6	Prospective	220	NICU	17,421	12,869	(53)
Mazhar *et al*, 2018	Saudi Arabia	12.0 d^b^	29.7	12.9	Prospective	138	NICU	583	248	(54)
Aamir *et al*, 2018	Pakistan	8.0±18.5 d^b^	52.1	33.4	Prospective	1,300	General ward	3,448	2,948	(55)
Landwehr *et al*, 2019	Australia	6.0±4.7 y^b^	54.0	1.6	Cross-sectional	190	General ward	1,160	644	(10)
Dornelles *et al*, 2019	Brazil	18.0 m^b^	44.6	27.0	Prospective	157	PICU	1,328	951	(56)
Kouti *et al*, 2019	Iran	34.0±4.4 w^b^	38.1	1.9	Cross-sectional	193	NICU	1,049	420	(57)
Tukayo *et al*, 2020	Indonesia	1.8 y^b^	71.5	79.0	Cross-sectional	200	General ward	1,961	1,557	(58)
Gidey *et al*, 2020	Ethiopia	0.0-28.0 d	67.6	23.6	Cross-sectional	122	NICU	366	334	(59)
García-López *et al*, 2020	Italy	9.0 m^b^	39.6	12.9	Cross-sectional	85	General ward	1,198	630	(60)
AlAzmi *et al*, 2021	Saudi Arabia	4.0 y^b^	39.4	-	Retrospective	326	General ward	865	341	(61)

^a^Median;

^b^mean. -, not available; NICU neonatal intensive care unit; PICU, pediatric intensive care unit; d, days; w, weeks; m, months; y, years.

## Data Availability

The data generated in the present study may be requested from the corresponding author.
