# Supplementary material for: Off‑label and unapproved pediatric drug utilization: A meta‑analysis
Source: Exp Ther Med. 2024 Aug 30;28(5):412. doi: 10.3892/etm.2024.12701 (PMC11391174; doi:10.3892/etm.2024.12701)

Figure S1. Sensitivity analysis indicating that the pooled prevalence remains notably stable upon exclusion of each study.

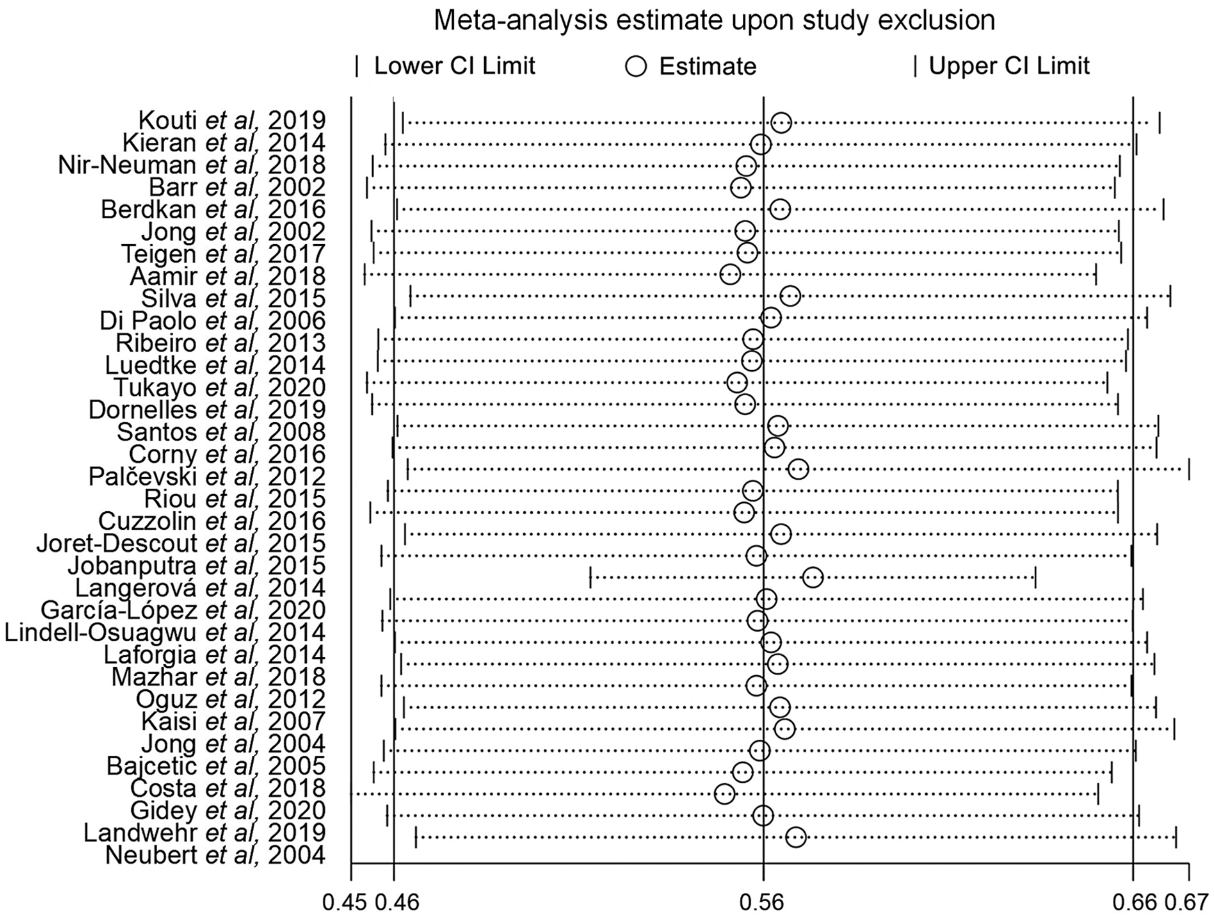

Figure S2. Sensitivity analysis to assess the impact of excluding studies that had a small sample size of prescriptions.

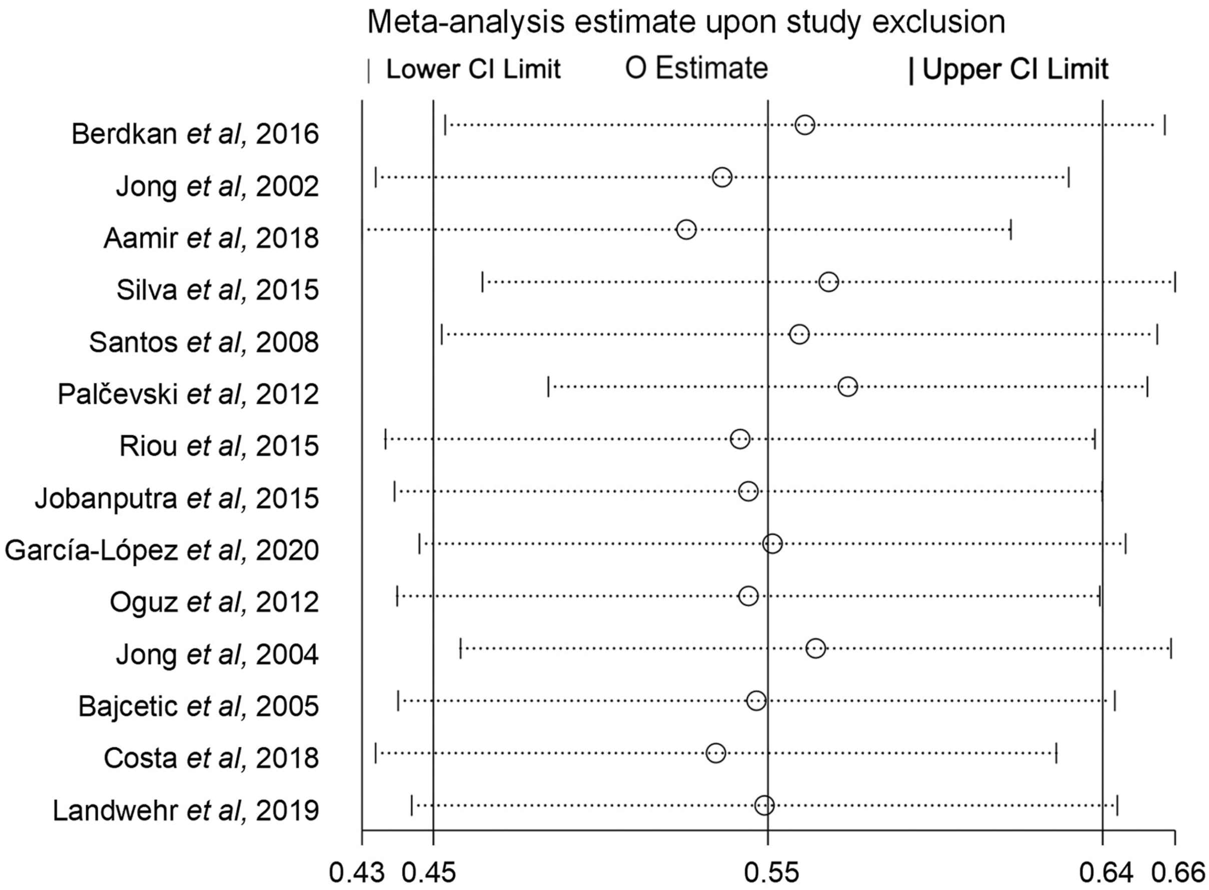

Figure S3. Forest plot of prevalence of off-label and unlicensed prescriptions in a pediatric population after excluding studies that had a small sample size of prescriptions.

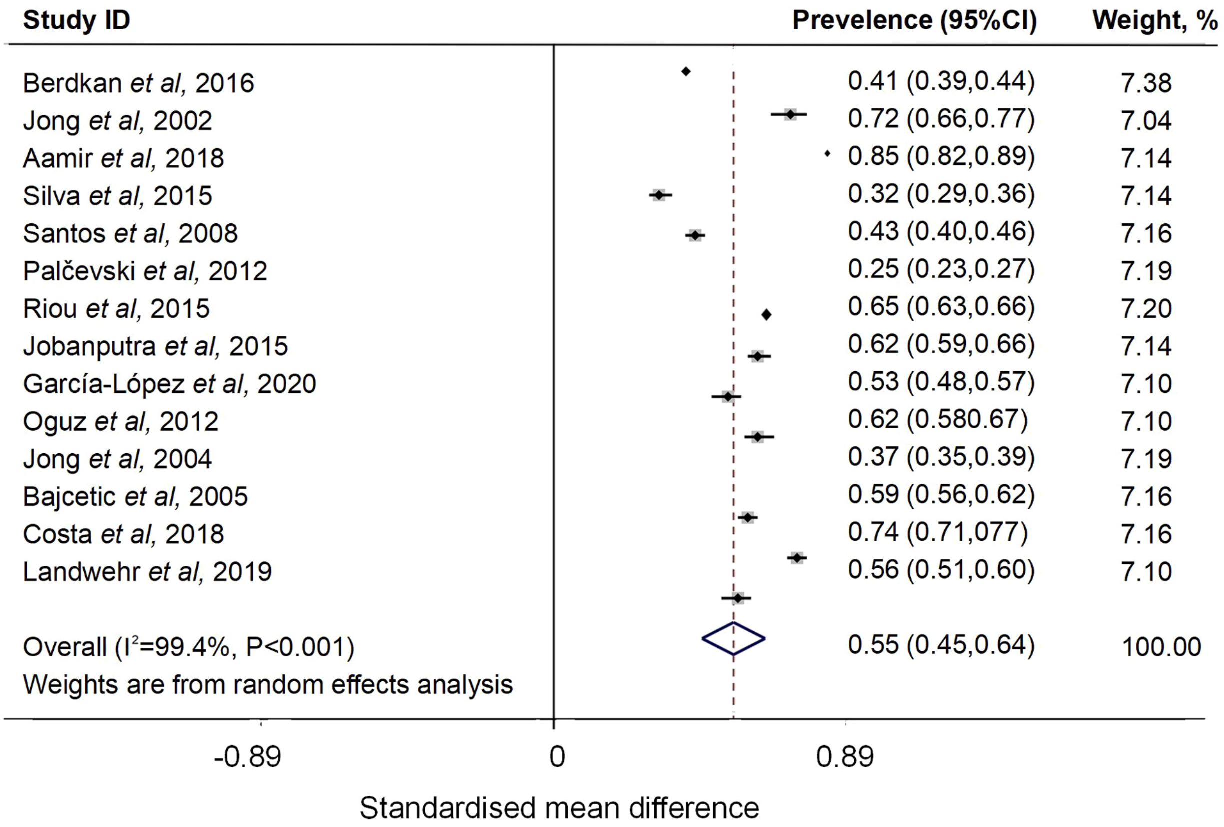

Figure S4. Regression-based Egger's test examining off-label and unlicensed prescriptions.

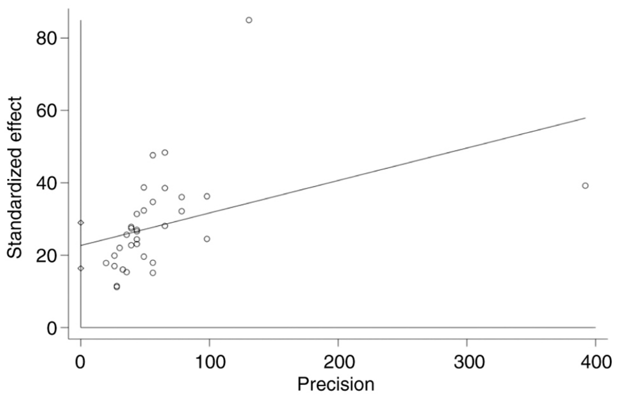

Supplement: Sensitivity analysis indicating that the pooled prevalence remains notably stable upon exclusion of each study. [file Supplementary_Data1.pdf]
