# Supplementary material for: Off‑label and unapproved pediatric drug utilization: A meta‑analysis
Source: Exp Ther Med. 2024 Aug 30;28(5):412. doi: 10.3892/etm.2024.12701 (PMC11391174; doi:10.3892/etm.2024.12701)
Supplement: Quality assessment of included articles using the Joanna Briggs Institute critical assessment checklist. [file Supplementary_Data2.pdf]

Table SI. Quality assessment of included articles using the Joanna Briggs Institute critical assessment checklist.

First author, year	1	2	3	4	5	6	7	8	9	Overall quality	(Ref.)
Turner *et al,* 1999	Yes	NA	Yes	Yes	Yes	Yes	Yes	Yes	NA	High	(19)
Conroy *et al,* 1999	Yes	NA	Yes	Yes	Yes	Yes	Yes	Yes	NA	High	(20)
Pandofini *et al,* 2002	Yes	NA	Yes	Yes	Yes	Yes	Yes	Yes	NA	High	(21)
Barr *et al,* 2002	No	NA	Yes	Yes	Yes	Yes	Yes	Yes	NA	High	(22)
Jong *et al,* 2002	Yes	NA	No	Yes	Yes	Yes	Yes	Yes	NA	High	(23)
Conroy *et al,* 2003	Yes	NA	Yes	Yes	Yes	Yes	Yes	Yes	NA	High	(24)
Jong *et al,* 2004	Yes	NA	No	Yes	Yes	Yes	Yes	Yes	NA	High	(25)
Neubert *et al,* 2004	Yes	NA	Yes	Yes	Yes	Yes	Yes	Yes	NA	High	(26)
Bajcetic *et al,* 2005	Yes	NA	No	Yes	Yes	Yes	Yes	Yes	NA	High	(27)
Di Paolo *et al,* 2006	Yes	NA	No	Yes	Yes	Yes	Yes	Yes	NA	High	(28)
Kaisi *et al,* 2007	Yes	NA	Yes	Yes	Yes	Yes	Yes	Yes	NA	High	(29)
Santos *et al,* 2008	Yes	NA	Yes	Yes	Yes	Yes	Yes	Yes	NA	High	(30)
Bavdekar *et al,* 2009	No	NA	Yes	Yes	Yes	Yes	Yes	Yes	NA	High	(31)
Lass *et al,* 2011	Yes	NA	Yes	Yes	Yes	Yes	Yes	Yes	NA	High	(32)
Palčevski *et al,* 2012	Yes	NA	No	Yes	Yes	Yes	Yes	Yes	NA	High	(33)
Oguz *et al,* 2012	Yes	NA	No	Yes	Yes	Yes	Yes	Yes	NA	High	(34)
Ballard *et al,* 2013	No	NA	No	Yes	Yes	Yes	Yes	Yes	NA	High	(35)
Kieran *et al,* 2014	No	NA	Yes	Yes	Yes	Yes	Yes	Yes	NA	High	(36)
Silva *et al,* 2015	Yes	NA	Yes	Yes	Yes	Yes	Yes	Yes	NA	High	(37)
Lee *et al,* 2013	Yes	NA	No	Yes	Yes	Yes	Yes	Yes	NA	High	(38)
Ribeiro *et al,* 2013	No	NA	Yes	Yes	Yes	Yes	Yes	Yes	NA	High	(39)
Lindell-Osuagwu *et al,* 2014	No	NA	No	Yes	Yes	Yes	Yes	Yes	NA	High	(40)
Laforgia *et al,* 2014	Yes	NA	Yes	Yes	Yes	Yes	Yes	Yes	NA	High	(41)
Langerová *et al,* 2014	No	NA	No	Yes	Yes	Yes	Yes	Yes	NA	High	(42)
Luedtke *et al,* 2014	No	NA	No	Yes	Yes	Yes	Yes	Yes	NA	High	(43)
Riou *et al,* 2015	No	NA	Yes	Yes	Yes	Yes	Yes	Yes	NA	High	(44)
Joret-Descout *et al,* 2015	Yes	NA	No	Yes	Yes	Yes	Yes	Yes	NA	High	(45)
Jobanputra *et al,* 2015	Yes	NA	Yes	Yes	Yes	Yes	Yes	Yes	NA	High	(46)
Berdkan *et al,* 2016	Yes	NA	Yes	Yes	Yes	Yes	Yes	Yes	NA	High	(47)
Cuzzolin *et al,* 2016	No	NA	No	Yes	Yes	Yes	Yes	Yes	NA	High	(48)
Corny *et al,* 2016	Yes	NA	Yes	Yes	Yes	Yes	Yes	Yes	NA	High	(49)
Ramadaniati *et al,* 2017	Yes	NA	Yes	Yes	Yes	Yes	Yes	Yes	NA	High	(50)
Tefera *et al,* 2017	Yes	NA	No	Yes	Yes	Yes	Yes	Yes	NA	High	(51)
Teigen *et al,* 2017	Yes	NA	Yes	Yes	Yes	Yes	Yes	Yes	NA	High	(52)
Nir-Neuman *et al,* 2018	No	NA	Yes	Yes	Yes	Yes	Yes	Yes	NA	High	(9)
Costa *et al,* 2018	No	NA	Yes	Yes	Yes	Yes	Yes	Yes	NA	High	(53)
Mazhar *et al,* 2018	No	NA	No	Yes	Yes	Yes	Yes	Yes	NA	High	(54)
Aamir *et al,* 2018	Yes	NA	Yes	Yes	Yes	Yes	Yes	Yes	NA	High	(55)
Landwehr *et al,* 2019	Yes	NA	Yes	Yes	Yes	Yes	Yes	Yes	NA	High	(10)
Dornelles *et al,* 2019	Yes	NA	Yes	Yes	Yes	Yes	Yes	Yes	NA	High	(56)
Kouti *et al,* 2019	No	NA	Yes	Yes	Yes	Yes	Yes	Yes	NA	High	(57)
Tukayo *et al,* 2020	Yes	NA	Yes	Yes	Yes	Yes	Yes	Yes	NA	High	(58)
Gidey *et al,* 2020	No	NA	No	Yes	Yes	Yes	Yes	Yes	NA	High	(59)
García-López *et al,* 2020	Yes	NA	Yes	Yes	Yes	Yes	Yes	Yes	NA	High	(60)
AlAzmi *et al,* 2021	Yes	NA	No	Yes	Yes	Yes	Yes	Yes	NA	High	(61)

1, Was the sample frame appropriate to address the target population?; 2, were study participants recruited in an appropriate way?; 3, was the sample size adequate?; 4, were study subjects and setting described in detail?; 5, was data analysis conducted with sufficient coverage of the identified sample?; 6, were valid methods used for identification of the condition?; 7, was the condition measured in a standard, reliable way for all participants?; 8, was there appropriate statistical analysis?; 9, was the response rate adequate and if not, was the low response rate managed appropriately?; NA, not applicable.

Table SII. Subgroup analysis by study design and continent.

A, Overall pooled prevalence of off-label and unlicensed drug use

Subgroup	No. of studies	Prevalence	95% CI	Heterogeneity, %	P-value
Study design					
Cross-sectional	12	0.54	0.43-0.65	98.9	<0.001
Prospective	18	0.60	0.54-0.67	98.2	<0.001
Retrospective	4	0.38	0.17-0.59	99.8	<0.001
Continent					
Europe	18	0.49	0.36-0.62	99.8	<0.001
Asia	8	0.65	0.52-0.78	98.8	<0.001
North America	2	0.56	0.36-0.76	98.6	<0.001
South America	3	0.63	0.42-0.84	99.1	<0.001
Africa	2	0.66	0.17-1.15	98.4	0.008
Australia	1	0.56	0.51-0.60	-	<0.001

B, Overall pooled prevalence of off-label drug use among pediatric patients

Subgroup	No. of studies	Prevalence	95% CI	Heterogeneity, %	P-value
Study design					
Cross-sectional	5	0.43	0.21-0.64	99.0	<0.001
Prospective	16	0.52	0.45-0.58	99.7	<0.001
Retrospective	4	0.27	0.14-0.41	99.6	<0.001
Continent					
Europe	12	0.42	0.29-0.55	99.8	<0.001
Asia	8	0.52	0.42-0.63	98.7	<0.001
North America	1	0.38	0.36-0.41	-	<0.001
South America	3	0.45	0.39-0.50	90.0	<0.001
Australia	1	0.54	0.50-0.58	-	<0.001

C, Overall pooled prevalence of unlicensed medication use among pediatric patients

Subgroup	No. of studies	Prevalence	95% CI	Heterogeneity, %	P-value
Study design					
Cross-sectional	12	0.16	0.10-0.22	98.9	<0.001
Prospective	17	0.22	0.16-0.27	98.2	<0.001
Retrospective	5	0.11	0.03-0.19	99.8	0.011
Continent					
Europe	17	0.12	0.09-0.15	99.1	<0.001
Asia	10	0.25	0.16-0.34	99.4	<0.001
North America	1	0.08	0.06-0.09	-	<0.001
South America	3	0.36	0.20-0.51	98.9	<0.001
Africa	2	0.27	0.20-0.34	67.6	<0.001
Australia	1	0.02	0.02-0.03	-	<0.001
